# The Assessment of Visual Fields in Infants Using Saccadic Vector Optokinetic Perimetry (SVOP): A Feasibility Study

**DOI:** 10.1167/tvst.10.3.14

**Published:** 2021-03-12

**Authors:** Antonios Perperidis, Alice D. McTrusty, Lorraine A. Cameron, Ian C. Murray, Harry M. Brash, Brian W. Fleck, Robert A. Minns, Andrew J. Tatham

**Affiliations:** 1 Department of Child Life and Health, University of Edinburgh, Edinburgh, UK; 2 Centre for Clinical Brain Sciences, University of Edinburgh, Edinburgh, UK; 3 Department of Vision Sciences, Glasgow Caledonian University, Glasgow, UK; 4 Royal Hospital for Sick Children, Edinburgh, UK; 5 Princess Alexandra Eye Pavilion, Edinburgh, UK

**Keywords:** infants, vision, perimetry, eye tracking, medical devices

## Abstract

**Purpose:**

To examine the feasibility of saccadic vector optokinetic perimetry (SVOP), an automated eye tracking perimeter, as a tool for visual field (VF) assessment in infants.

**Methods:**

Thirteen healthy infants aged between 3.5 and 12.0 months were tested binocularly using an adapted SVOP protocol. SVOP uses eye tracking technology to measure gaze responses to stimuli presented on a computer screen. Modifications of SVOP for testing infants included adjusting the fixation target to display a short animation, increasing the stimulus size to equivalent to Goldmann V, and introducing a tiered test pattern strategy. Binocular, single-quadrant confrontation VF testing and Keeler preferential looking cards visual acuity testing was also performed.

**Results:**

Using multiple test attempts when required, all but the youngest infant (12 of 13 [92.3%]) successfully completed a 4-point screening test. Seven infants (53.8%) successfully completed the 12-point test, four (30.8%) successfully completed the 20-point test, and three (23.1%) successfully completed the 40-point test. The effect of multiple test attempts and the complexity of the test pattern (number of test points) on performance was investigated, including test completion rate, percentage of correctly seen stimuli, and average time per tested stimulus.

**Conclusions:**

The modified SVOP test strategy allowed successful assessment of binocular VFs in healthy infants. Future data collection from larger cohorts of infants is needed to derive normative limits of detection and assess accuracy in detecting and monitoring infant VF abnormalities.

**Translational Relevance:**

Eye tracking perimetry may provide a useful method of automated VF assessment in infants.

## Introduction

Infants may have congenital or acquired defects of the visual field (VF) owing to a wide range of neurologic and ocular conditions, including brain tumours,^1^^,^^2^ elevated intracranial pressure,^3^ and neonatal hypoxic–ischemic encephalopathy.^4^ Ocular conditions potentially affecting the VF in infancy include congenital conditions such as coloboma. The ability to detect and measure VF defects in infancy would aid in the diagnosis, monitoring, and understanding of the impact of ocular and neurologic diseases on a child's visual function; however, the present methods of assessing VFs in infants are limited.

**Figure 1.**
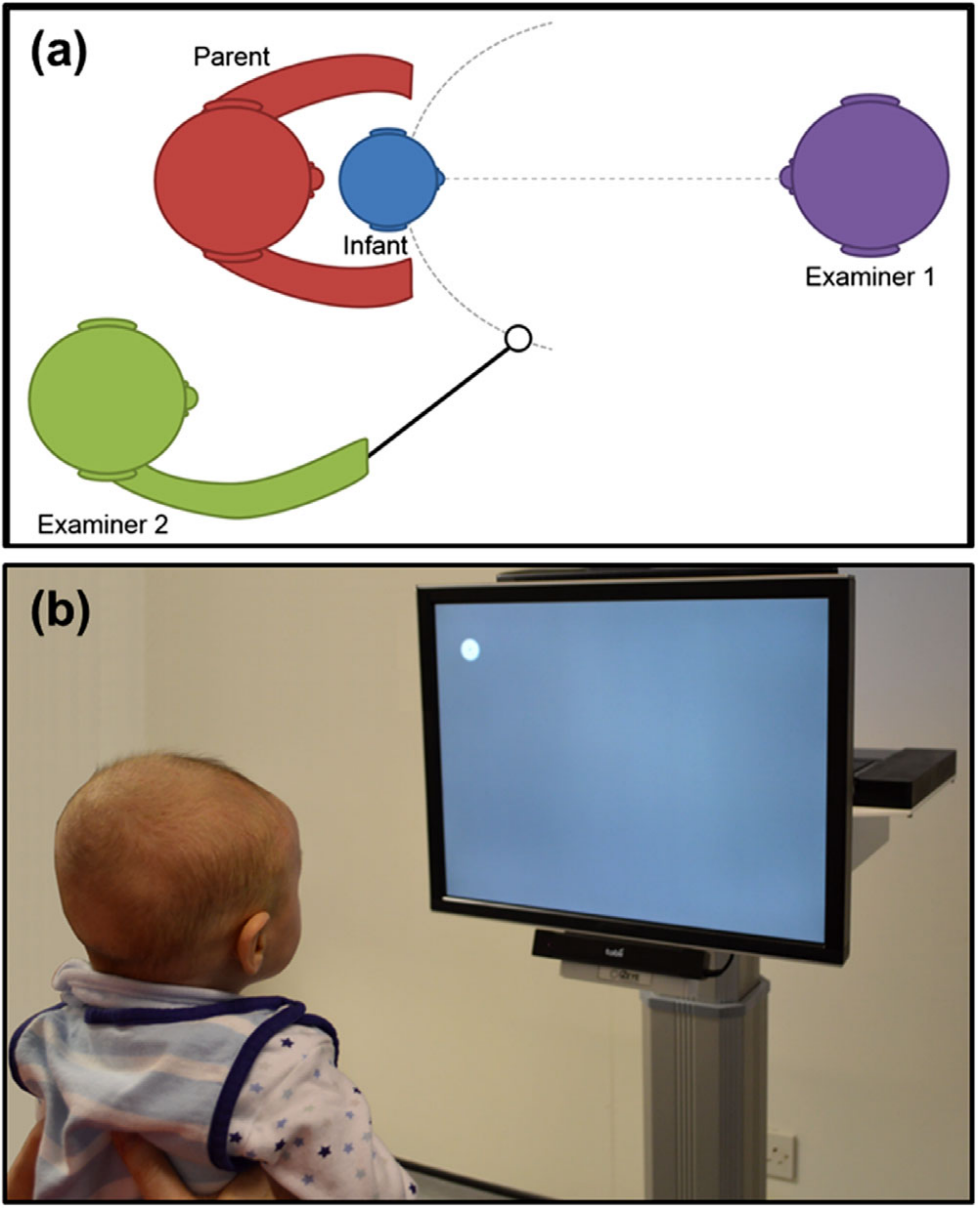
(**a**) Diagram illustrating a confrontational VF test. (**b**) An infant performing a threshold SVOP test with stimulus displayed at the top left corner of the display. The eye tracker is positioned beneath the patient LCD display monitor, while the device is controlled by an operator PC (behind patient monitor).

In adults, VFs can be assessed using automated static or manual kinetic perimetry. Although children older than 5 to 6 years of age can often perform perimetry reasonably well, perimetry requires sustained concentration and reliability is higher in older children and adults. Children aged 5 to 6 years have been shown to have higher rates of false positives and false negatives and larger intragroup variability compared with 7- to 8-year-old children and adults.^5^^–^^7^ Consequently, the assessment of VFs in younger children and infants has been restricted to predominantly manual, operator-driven methods such as confrontation testing, which is imprecise and operator dependent (Fig. 1a).^8^^–^^10^

Several investigators have attempted to improve VF testing in infants. Schwartz et al.^11^ described a modified manual kinetic perimeter that was used to assess VFs in infants. White Styrofoam spheres were used as stimuli, introduced along eight directions from a central fixation target. Infants were found to have smaller but similarly shaped VFs compared with adults. Lewis and Maurer^12^ evaluated VFs in infants up to 6 months of age, but used a method using a static flashing light stimulus, whereas Dobson et al.^13^ compared static and hybrid static-kinetic perimetry using LEDs and kinetic perimetry using Styrofoam spheres in a study of 180 normal children. They reported that kinetic perimetry resulted in larger, more adult-like VFs, which approached adult levels at around 17 months, compared with 30 months for static perimetry. Other approaches have included the Behavioral Visual Field screening test.^14^ The Behavioral Visual Field is a type of kinetic perimeter that measures VFs by observing responses to a stimulus on a graded arc that moves from the periphery to the center of the VF along different meridians. Koenraads et al.^15^ reported that 56% of infants younger than 1 year of age were able to produce reliable tests, increasing to 71% in those between 1 and 3 years of age, and 75% of those 2 years and older. Seventy-five percent of children were found to have consistent results on repeat testing.^15^

These studies demonstrate that manual, expert operator-driven approaches can yield valuable and detailed VF information. Nevertheless, manual VF assessments depend on operator experience and require a prolonged test duration. Satgunam et al.^16^ developed and tested a purpose-built pediatric perimeter that quantified VF extent and reaction time. The device was a computerized adaptation of a perimetric technique developed for infants,^17^ consisting of a hemispherical dome with LED positioned along 24 equidistant meridians 60 cm from the infant. A camera and fixation lights were positioned at the center of the dome and the reactions of the infants to known stimuli were recorded and manually analyzed by an operator. The recording of the reactions enabled verification of the decision, decreasing potential operator bias.

As with adults, young infants have an innate tendency to look toward objects appearing in their peripheral vision. A number of studies have attempted to use this tendency through the use of noncontact eye tracking technology. Franchak et al.^18^ used a head-mounted eye tracking device in an attempt to record and analyze the gaze responses of young children to “natural stimuli” during free play with mothers. Jones et al.^19^^,^^20^ described the use of an eye tracker to assess the binocular visual acuity in infants by displaying a sine-wave grading stimulus 8° off the fixation point. The infants’ gaze responses to the stimulus were recorded through eye tracking and an automated decision was made whether the stimulus was seen.^19^^,^^20^

Our group has described a system termed saccadic vector optokinetic perimetry (SVOP), which has been shown to be useful for assessment of VFs in children with localized cerebral abnormalities^1^^,^^21^^–^^23^ and adults with glaucoma.^23^^–^^26^ SVOP uses eye tracking to quantify refixation saccadic eye movements that occur in response to stimuli presented on a computerized screen. It does not require a chin rest or need the patient to press a response button. The software automatically adjusts the size and position of the stimulus allowing the patient to move their head freely during testing. A stimulus is registered as seen if there is a saccadic eye movement toward the stimulus within a prespecified time of first presentation (Fig. 1b). The aims of this study were to (i) propose a number of adaptations to the previously reported SVOP suprathreshold testing protocol,^1^^,^^21^^–^^23^ (ii) establish that the adapted SVOP can detect and assess infant eye gaze responses to VF stimuli, and (iii) identify limitations and then propose future technical developments necessary to create an effective clinical tool.

## Methods

Thirteen infants (eight males and five females) with a mean age of 8.8 ± 3.8 months, (range, 3.5–12.0 months) were tested using SVOP at the Clinical Research Facility at the Department of Child Life and Health, University of Edinburgh (Edinburgh, UK) and the Department of Vision Sciences at Glasgow Caledonian University (Glasgow, UK). All infants were neuro-developmentally normal, with no significant medical or ophthalmic diagnoses, nor any parental concerns related to visual symptoms. The study adhered to the tenets of the Declaration of Helsinki and was approved by the Lothian Research Ethics Committee (REC reference number 13/SS/0045). All testing was done after fully informed parental consent.

### Confrontation VF Testing and Visual Acuity Testing

Before SVOP testing, all infants had binocular, single-quadrant confrontation VF testing,^8^^–^^10^ and visual acuity testing^27^^,^^28^ with Keeler (Keeler Ltd., Windsor, Berkshire, UK) preferential looking cards. During confrontation testing, the infant was positioned on the parent's lap with one of the operators facing and engaging the infant at an approximate distance of 1 m and at the same eye level as the infant, ensuring fixation on the operator's eyes (bridge of the nose). The second operator was positioned behind the parent (and infant) and presented a toy in each VF quadrant. The stimulus was deemed as “seen” when the operator facing the infant observed a rapid eye and/or head movement toward the presented stimulus.

During visual acuity testing, the infant was positioned on the parent's lap with the operator facing the infant at an approximate distance of 35 cm. The operator presented preferential looking cards, where one side contained the grating test area, and the other side contained a uniform grey test area of equivalent mean luminance. Starting with a lower spatial frequency grating, the infant's reaction was observed through the available small peephole in the center of the cards. The decision on whether the grating target was seen was based on a range of observed cues (such as fixation and eye and head movements) with the greatest spatial frequency grating that yielded a consistent positive response on three occasions, indicating an estimate of the infant's visual acuity.

### Saccadic Vector Optokinetic Perimetry

**Figure 2.**
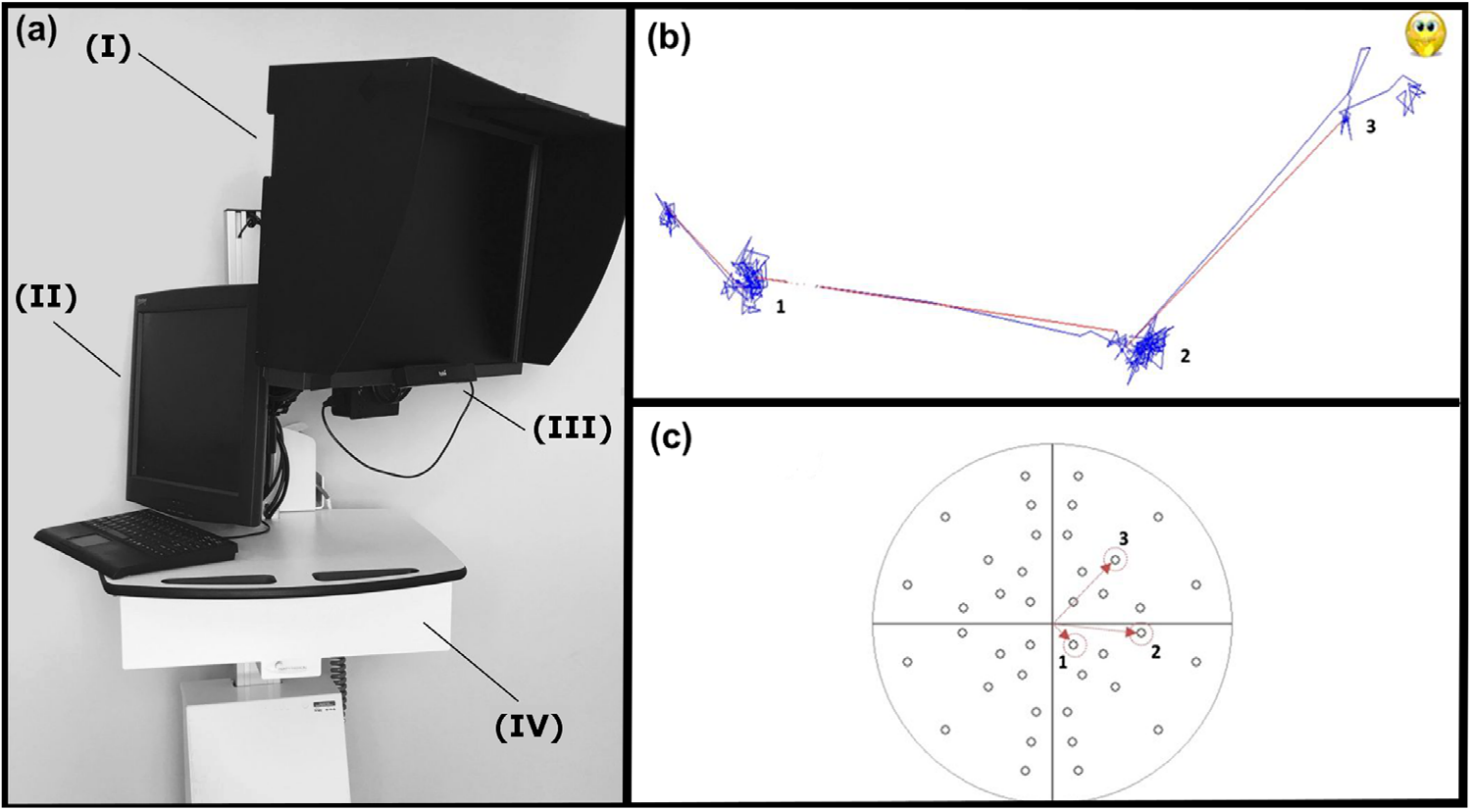
(**a**) The SVOP system components. (I) subject display, (II) examiner display, (III) eye tracker, and (IV) height adjustable surface housing the personal computer. (**b**, **c**) Example of eye gaze movements for three different VF points which were all “seen” and their associated position within a normal VF plot. (**b**) Blue lines represented eye gaze movements made every 20 ms. Red lines represented a change in fixation (saccade) detected by SVOP. (**c**) A normal VF plot (with all points “seen”). The three numbered points (highlighted with red arrows and circles) corresponded to the fixation changes numbered in (**b**).

The SVOP system consists of four main components (Fig. 2a): (i) the subject display, where the fixation target and stimuli are presented, (ii) the eye tracker, which records the subject's gaze responses, (iii) the operator display, which enables setting up and monitoring the progress of the test, and (iv) a personal computer to connect and control all the components as well as run the test and all associated algorithms. In the SVOP version used throughout this study, a 20” DELL 2005FPW (Dell Inc., Austin, TX) LCD was used as the subject display along with a Tobii IS-1 (Tobii Technology, Stockholm, Sweden) 40-Hz noncontact eye tracker. The display was calibrated at the beginning of the study, ensuring a uniform luminance response across the whole field of view and therefore an accurate representation of the both the background and stimuli luminance levels.^29^ Table 1 provides the technical specifications of the individual components.

**Table 1.** Display (20-Inch Dell 2005FPW) and Eye Tracker (Tobii IS-1) Technical Specifications

20-Inch Dell 2005FPW	
Dimensions (W × D × H)	472 **×** 229 **×** 389 mm
Aspect ratio	16:10
Native resolution	1680 **×** 1050 at 60HZ
Contrast ratio	600:1
Maximum brightness	300 cd/m^2^
Refresh rate (horizontal, vertical)	83 Hz, 75 Hz
Tobii IS-1	
Head movement allowance (W × D × H)	400 **×** 300 **×** 400 mm
Distance of optics to eye	Ideally 0.6m
Maximum sampling rate	40Hz
Accuracy	<0.5°

The eye tracker provided real-time data on the three-dimensional eye position relative to the display, and the point of gaze on the display screen. This information allowed for the real-time (i) estimation and adjustment of the screen coordinates and size (in pixels) of VF stimuli, facilitating free head movement during the testing without the use of a chin rest or other restrictive devices, (ii) establishment of whether fixation on a target has occurred, and (iii) assessment of eye gaze responses to VF stimuli (presented only after establishing fixation). The only task required of a subject was to respond to a peripheral stimulus (if seen), which then became the fixation target for the next peripheral test point. An algorithm determined whether a VF stimulus was perceived based on the direction and amplitude of a subject's eye gaze response. Detection limits were derived using normative data, both monocular and binocular previously obtained from 38 volunteers (20 adults and 18 children). The SVOP operation and the decision algorithm have been described in detail previously.^22^

### SVOP Test Procedure

Infants were positioned, seated on the lap of their parent, centrally in front of (approximately 60 cm away) the subject display (Fig. 1b). If necessary, the subject display was adjusted to ensure optimal positioning by using real-time feedback on the eye position relative to the subject display provided by the eye tracker. Before each test, a short (<60-s) calibration procedure was performed requiring the infants to follow a visual stimulus (cartoon character) with their gaze, to five different screen locations, presented in random order, with a short animation to hold the attention of the infant. This procedure allowed the geometric characteristics of the infant's eyes to be determined, therefore allowing accurate, subject-specific gaze position data. Each infant required a single successful calibration per visit.

**Figure 3.**
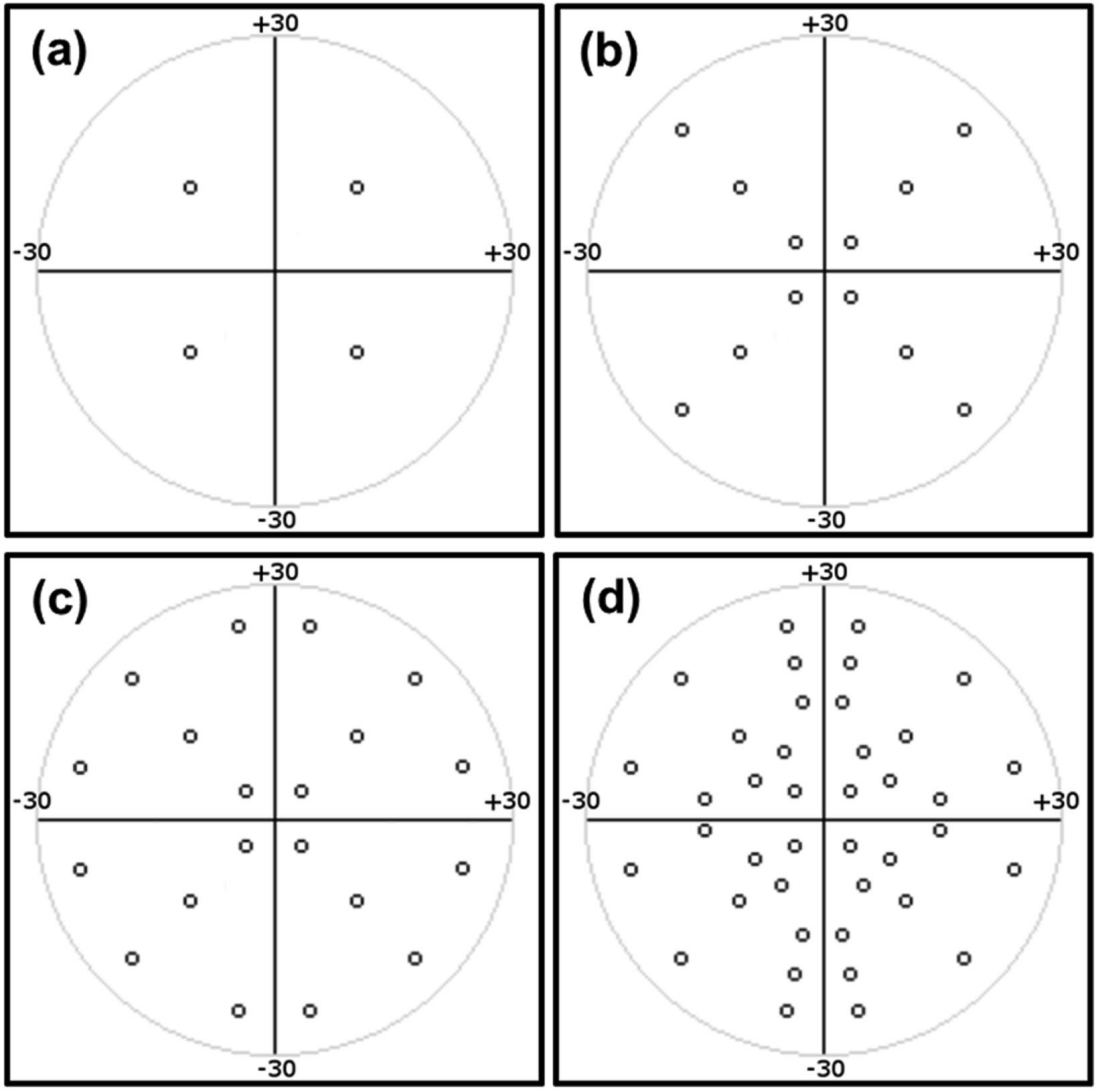
The 4-, 12-, 20-, and 40-point test patterns. The 40-point pattern is equivalent to the HFA C-40 pattern including points at 5°, 10°, 20°, and to 25° of eccentricity.

All tests were binocular. A high contrast target on the infant's LCD display was used to fixate the infant's gaze to a location. Once fixation was automatically verified through the real-time gaze data, the fixation target was removed and a bright, suprathreshold test stimulus was displayed against a dark background (luminance of 137 and 10 cd/m^2^, respectively) for 200 ms. The stimulus was positioned at a predetermined VF location relative to the current fixation position to assess the points of a predetermined test pattern (Fig. 3). A 200-ms stimulus duration was chosen as a commonly used option for VF in children^1^^,^^6^ across a range of devices, including Humphrey Field Analyzer (HFA) and novel platforms.^30^ The stimulus and background brightness levels used were equivalent to 14 dB. The gaze response of the infant to the stimulus was recorded by the eye tracker and quantified by the SVOP software, which decided whether the stimulus was seen or unseen. Figures 2b and 2c provide examples of eye gaze movements for three different VF points that were all “seen” and their associated position within a normal VF plot. A successful fixation at the stimulus test point then became the fixation starting point for the next test stimulus. The process was repeated until all the points in the VF test-pattern were tested. To accommodate for the younger age group (i.e., infants) of the subjects in this study, several adaptations to the previously reported SVOP suprathreshold screening testing protocol^21^^,^^23^ were introduced.

### SVOP Modifications

#### Stimulus Size

Preliminary investigation using six infants (five males, one female; mean age, 10.0 ± 1.7 months; range, 8–12 months) with stimulus sizes equivalent to Goldmann III, IV, and V was performed, to determine the optimum stimulus size for infants. These infants were independent of the cohort of 13 infants reported in this study. All stimuli were circular spots with angular diameters of 0.43°, 0.86°, and 1,72° for Goldmann III, IV, and V, respectively. Test patterns of up to 40 points were used. For stimulus size of Goldmann V, 565 points were presented (cumulative) and 487 of these were seen (86.2%). For stimulus sizes of Goldmann IV and III, a lower percentage of shown points were seen (78.3% and 71.7%, respectively). Consequently, because the test is suprathreshold screening, the larger Goldmann V stimulus was selected as the stimulus size for the remainder of the study.

#### Fixation Target

Consecutive SVOP versions have used a range of strategies to ensure initial fixation at a desirable location within the subject LCD display. Typically, fixation has been achieved and maintained with a recursive resizing of a circular object, centered at the fixation coordinates. When fixation is established (through eye tracking), the target is removed to display the VF test stimulus. In this study a short animation (with sound) was used to initially engage infants before presentation of each test stimulus.

#### Tiered Stimulus Order Approach

**Figure 4.**
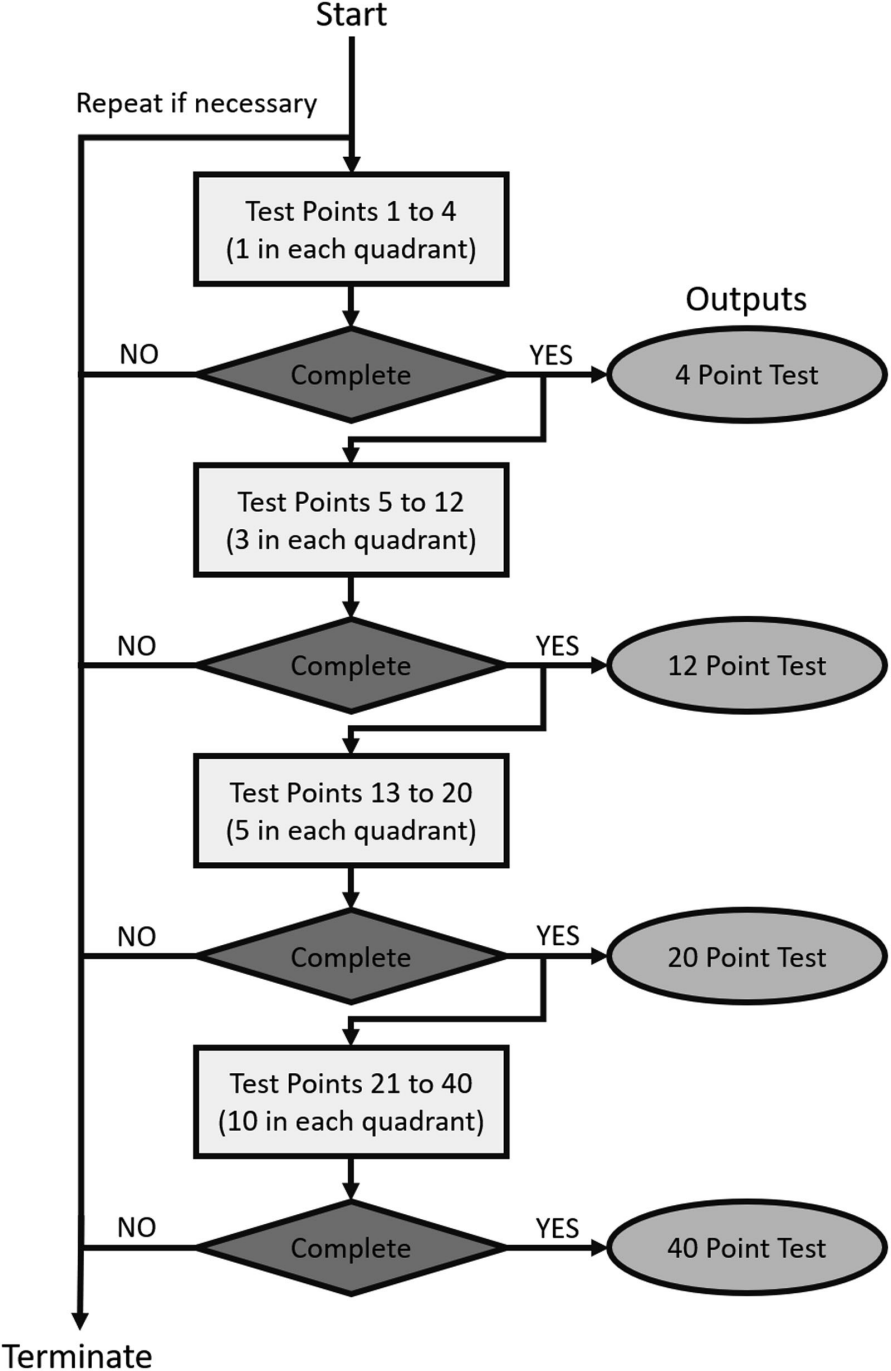
Flow diagram describing the tiered approach for testing infants, starting from a basic 4-point test (1 point in each quadrant) and progressively introducing points to a complete 40-point test pattern.

During a preliminary investigation using six infants (independent of the 13-infant cohort), it became apparent that the attention span of infants varied greatly. We therefore used a sequential tiered testing strategy where initially four locations, one in each quadrant, were tested. After these were tested, two additional points in each quadrant were automatically introduced (one stimulus at a time) resulting in a 12-point test pattern. Subsequently, two more stimuli in each quadrant were tested, resulting in a 20-point test, and so on, leading finally to the complete 40-point test. The final test pattern was equivalent to the Humphrey VF Analyzer C-40 screening test pattern, consisting of 40 points (with 10 points in each quadrant) arranged with eccentricity ranging between 5° and 25°. The C-40 pattern was chosen as a commonly used screening pattern, facilitating comparison. The 4-, 12-, and 20-point patterns were subsets of the C-40 (Fig. 3), and tiered testing proceeded to the next tier upon completion of the associated pattern (4, 12, and 20 points). With this progressive tiered system (Fig. 4), if the infant lost interest in the test, for example, after 9 points, we had a complete 4-point test, with 1 point in each quadrant. Similarly, if the infant lost interest after 15 points, we had a complete 12-point test, with 3 points in each quadrant. If the infant became disengaged or unhappy, the test was terminated. After a short break (approximately 5 minutes) the test was restarted. As a result of a software limitation, it was not possible to resume the test from the point of termination. Therefore, for any infant requiring a break, a new test had to be initiated. The duration of the break, the number of times a new test was initiated, and the decision on whether to proceed to the next tier or discontinue testing was made by the clinical operator. The reported test points consisted of the combination of points tested across the multiple tests.

### Data Analysis

SVOP records the saccadic responses (starting position, path, and terminal position) of the infants to each stimulus. Furthermore, SVOP provides a replay functionality, displaying the location of the tested stimulus and the associated saccadic responses (Fig. 2b). On completion of each test, data for the automated decisions on whether a stimulus was seen, the duration of each response, and the cumulative duration of the whole test were stored. An operator then investigated the recorded saccade responses to any apparently unseen points using the playback function, to verify whether an appropriate decision had been made by the automated algorithm. The decision was adjusted manually from unseen (negative) to seen (positive) if a saccade in the correct direction followed by fixation near yet marginally outside the current spatiotemporal window for detection was observed. The average time per stimulus for each test was estimated as *t*_stim_ = *T*_test_/*N*, the ratio of the associated full test duration *T*_test_ divided by the total number of points shown *N*.

## Results

**Figure 5.**
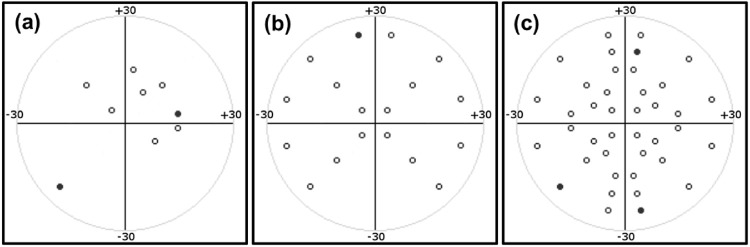
Characteristic examples of both complete (**b** and **c**) and incomplete tests (**a**). *White circles* indicate a point tested and seen by the subject (positives) and *black spots* indicate points tested but not seen by the subject (negatives). (**a**) Test performed before the tiered approach proposed in this paper with 7 points seen yet no standard test pattern being complete. (**b**) Example of a test terminated before reaching the 20-point pattern yielding a complete 12-point test. (**c**) Example of complete 40-point test with spurious points identified as unseen by current limits of detection and subsequently rectified through manual investigation.

**Figure 6.**
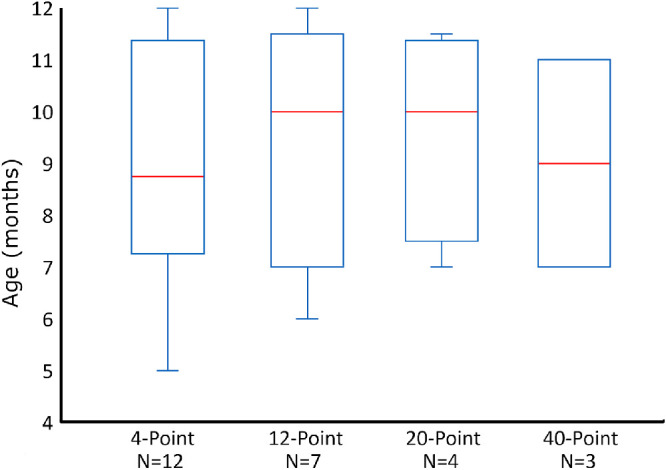
Age ranges in number of months for successfully completing SVOP tests of incremental test patterns sizes (Goldmann V stimulus). Number of infants (N) successfully completing each pattern size also provided. Box and whiskers plots were used to display results, with the central mark indicating the median, the bottom and top edges indicating the 25th and 75th percentiles respectively, and the whiskers extending to the most extreme values of the distribution.

The study included 13 infants (8 males and 5 females) with a mean age of 8.8 ± 3.8 months (range, 3.5–12.0 months). All infants tested had normal visual function with acuity ranging from 6/19 to 6/28, and positive responses to stimuli in all four VF quadrants. Using multiple test attempts when required, all but the youngest infant (12/13 [92.3%]) successfully completed a 4-point screening test (Table 2). Of the 12 infants who completed the 4-point test, the operator terminated the testing protocol for 3 infants at that point (Fig. 4), with 9 infants proceeding to the 12-point test. Seven infants (53.8%) successfully completed the 12-point test. Of the seven infants that completed the 12-point test, the operator terminated the testing protocol in two infants, with five infants proceeding to the 20-point test. Four infants (30.8%) successfully completed the 20-point test. Of the four infants who completed the 20-point test, the operator terminated testing in one infant and three infants (23.1%) proceeded and completed the 40-point test. The testing protocol was terminated when an infant became disengaged or unhappy. In two cases, multiple visits within a few days were required. A test was incomplete if an infant was entered into a testing tier but failed to complete the tier after multiple attempts. Examples of complete and incomplete SVOP tests are shown in Figure 5. All incomplete tests came from the two youngest infants, with a 3.5-month-old infant not completing either the 4-point or the 12-point test patterns, and a 5-month-old infant failing to complete the 12-point and 20-point test patterns (while coming close to both with 11/12 and 17/20 points seen, respectively). Figure 6 summarizes the age ranges of the infants successfully completing each test.

**Table 2.** Infants Performing 4-, 1-2, 20-, and 40-Point SVOP Test Patterns (Goldmann V Stimulus)

	4-Point Test	12-Point Test	20-Point Test	40-Point Test
No. of infants attempted the test	13	9	5	3
No. of infants successfully completing all points	12	7	4	3
Percent of attempting infants successfully completing the test	92.3	77.8	80.0	100.0
Percent of overall infants successfully completing the test	92.3	53.8	30.8	23.1

### Effect of Number of Test Attempts

**Figure 7.**
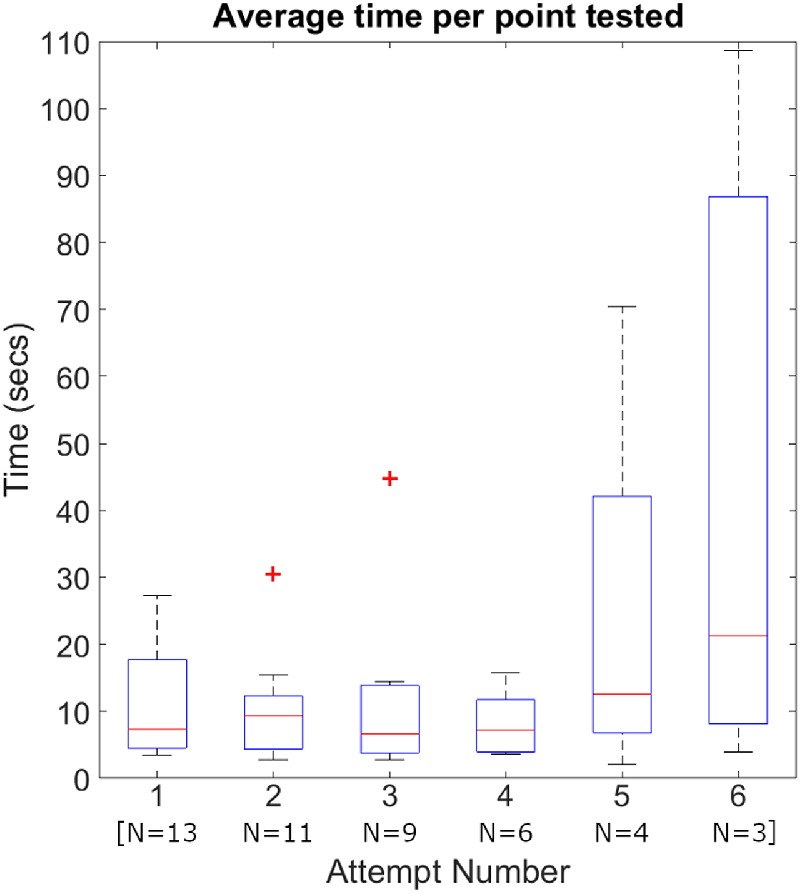
Distributions of average time per point tested, estimated as a ratio of number of points tested over the overall test duration, for increasing number of repeated test attempts. Reported durations do not include setup and calibration time. Box and whiskers plots were used to display results, with the central mark indicating the median, the bottom and top edges indicating the 25th and 75th percentiles, respectively, and the whiskers extending to the most extreme values of the distribution, not considering outliers. Outliers were plotted individually in each series (+).

A total of 46 test attempts were made by the 13 infants. Table 3 shows the number of attempts required to complete each tier of test. Table 4 shows the proportion of tests completed by increasing the number of test attempts. Performance decreased with more attempts. Table 4 also shows the percentages of seen stimuli by increasing number of test attempts. The total percentage of points seen was similar on the first, second, and third attempts; however, in those infants requiring more attempts, the number of points seen decreased, reaching only 60% and 69% seen for attempts five and six. Figure 7 illustrates the effect of increasing number of attempts on the average time per stimulus tested, estimated as a ratio of number of points tested over the overall test duration. At the fifth and sixth attempts, the duration per point increased. Three infants successfully completed a test at the first attempt, one a 4-point, one a 12-point, and one a 40-point test pattern. Contrastingly, a single infant had six attempts at the test yet did not manage to successfully complete any.

**Table 3.** Number of Attempts Per Test Completion for Incremental SVOP Test Patterns Sizes (Goldmann V Stimulus)

No. of Attempts Per Successfully Completed Test	4-Point Test	12-Point Test	20-Point Test	40-Point Test
Mean	2.4	1.9	1.8	1.7
Standard deviation	1.8	1.2	0.5	0.6
Minimum	1	1	1	1
Maximum	6	4	2	2

**Table 4.** Effect of Multiple Test Attempts on the Number of Tests Completed/Tests Performed and Seen Points Rates

	Attempt Number					
	1	2	3	4	5	6
No. of completed/performed	6/13	4/11	3/9	2/6	1/4	0/3
Percent of completed/performed	46.1	36.4	33.3	33.3	25.0	0.0
Percent of seen points	88.4	88.8	84.6	75.0	60.0	69.2

### Effect of Test Pattern Complexity

**Figure 8.**
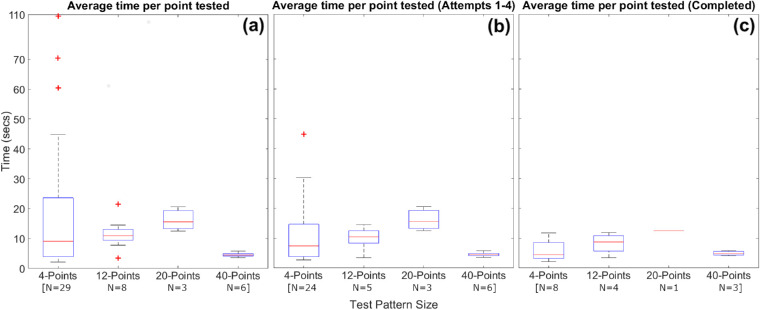
Boxplots illustrating the effect of increasing the complexity of the test pattern on the average time per stimulus tested across (**a**) all tests, (**b**) tests performed within the first four attempts, and (**c**) completed tests. Boxplots for each pattern size were derived using the number of the number (N) of tests (**a**, **b**) attempted and (**c**) completed. Time is estimated as a ratio of number of points tested over the overall test duration. The reported durations do not include setup and calibration time. Box and whiskers plots were used to display results, with the central mark indicating the median, the bottom and top edges indicating the 25th and 75th percentiles respectively, and the whiskers extending to the most extreme values of the distribution, not considering outliers. Outliers were plotted individually in each series (+).

Figure 8 illustrates the effect of increasing the complexity of the test pattern on the average time per stimulus tested. The boxplots for each pattern size were derived using the number of tests attempted in Figures 8a and 8b and completed in Figure 8c. Time was estimated as a ratio of the number of points tested over the overall test duration. Reported durations do not include setup and calibration time. Although the *t*_stim_ values varied greatly between 2.1 and 108.7 s, the majority of cases (>90%) demonstrated a *t*_stim_ of less than 25 s across all tests, a *t*_stim_ of less than 20 s across all tests in attempts one through four4, and a *t*_stim_ of less than 12.5 s across all completed tests.

### Manual Result Adjustment

Manual adjustment of unseen decisions through the available test playback functionality was necessary in 32% of the tests, readjusting 8% of the total points seen. In most cases, 1 or 2 points per test were readjusted with a maximum of 9 points observed at a 12-point and a 40-point test.

## Discussion

SVOP is suited for use in children because it does not require the child to place their chin on a rest and can detect whether a stimulus is seen using eye tracking rather than relying on pressing a response button. During this study, several modifications were introduced to tailor SVOP for use in infants, including using an animation as a fixation target, increasing the stimulus size to Goldmann size V equivalent, and using a tiered stimulus order approach. Using multiple test attempts when required, all but the youngest infant (12/13 [92.3%]) successfully completed a 4-point screening test, whereas 53.8%, 30.8%, and 23.1% of the infants successfully completed the 12-, 20-, and 40-point tests, respectively.

### SVOP Adaptations for Infants

By presenting stimuli in a tiered order, we were able to recover evenly distributed, predefined VF test patterns even in cases where early test termination was required. Figure 5a provides an example of an early terminated test where the tiered stimulus approach was not used. In the test, 7 points were seen by the subject, yet the points were unevenly spread across the VF. In contrast, Figure 5b provides an example of an early terminated test using the tiered approach. In this case, the 16 tested points were evenly spread, enabling a complete 12-point test pattern to be recovered. Although a complete 40-point test would facilitate a more comprehensive VF assessment, 12- and 20-point test patterns would be useful in the detection a range of VF defects, including hemianopia and quadrantanopia. The use of an animation was found to help maintain fixation and improve infants’ attention to the test, enabling more detailed test patterns to be completed successfully. The use of an animation had no significant effect on test duration, adding less than 1 s to the time spent on each tested stimulus.

### Test Duration and Number of Attempts

As shown in Figure 8, the average time per tested stimulus appeared to increase with the number of points tested between the 4-point to 20-point tests. The increase in time largely occurred owing to interruptions during the test owing to the larger window of opportunity for infants to become distracted when more points are assessed. Yet, for the infants successfully completing the 40-point test patterns, there was a substantial decrease in the average time per tested stimulus, as well as a decrease in the number of attempts required per completed tests (Table 3). This observation is indicative of the effect of an infant's engagement with the test-on-test performance. Infants highly engaged with the test completed large test patterns more quickly and with fewer repetitions. Infants not sufficiently engaged with the SVOP required repeated test attempts to generate a successfully completed test pattern. Eighty-five percent of infants required more than one attempt, most ranging between two and six attempts. As illustrated in Table 4 and Figure 7, the early repeated attempts did not seem to have a detrimental effect on performance, with only moderate changes in the number of attempted tests, completed tests, the average time per tested stimulus, and the percentage of points seen. For larger numbers (four or more) of repeated attempts, performance dropped considerably, across all examined metrics. Consequently, a large number of repeated attempts is not recommended as a means of generating reliable results.

### Limits of Detection

Based on the existing limits of detection, there were approximately 8% misclassified points that were manually adjusted based on the recorded gaze path response. During SVOP screening tests in normal infants, all misclassified points are likely to be false negatives (“unseen” in an area of normal VF), and review of responses confirmed that at least some were. The current detection limits used data from children older than 2 years,^22^ giving rise to misclassified false-negatives points (unseen) in infants. Further work is needed to adjust detection limits for use in infants. Those unseen responses not accounted for in this way were probably caused by a loss of attention. Future work with repeated testing will be required to confirm this supposition. The SVOP algorithm used stringent limits of detection, requiring a saccadic response (not just random saccadic motion) within a predetermined magnitude and direction over a short time interval (< 1 s) after the test stimulus was displayed. Even if the limits are relaxed for use in infants, we believe this approach almost completely precludes the occurrence of false positives (“seen” in an area of abnormal VF). Our previous validation and repeatability work in children with known pathology^1^^,^^21^ and in adults with glaucoma ^25^^,^^26^ confirms that this is highly likely.

### Limitations and Future Work

This study provides a first step toward using SVOP for the assessment of VFs in infancy.^31^^,^^32^ The study was limited by its small sample size and the inclusion of only healthy infants. In the future, larger cohorts of healthy infants as well as infants with ocular and neurologic diseases affecting the visual pathways will be tested. Such data may facilitate (i) the derivation of new detection limits for seen stimuli based on infant normative data, (ii) the correlation between age and performance characteristics, and (iii) a more complete analysis of the ability of SVOP to assess VFs in infancy. Establishing the clinical value of SVOP in infants will also include the ability to repeatedly detect and monitor progress of VF defects over time. All testing in this study was binocular. Future work will include attempts to perform uniocular testing, using an infrared filter lens over one eye to allow continued tracking of both eyes, as we routinely use in older children^22^ and in adults.^24^^,^^25^ However, binocular VF testing in infants with neurologic disease is likely to produce useful diagnostic and functional information.

In this study, a stimulus duration of 200 ms (followed by an allowed 1-s saccade response time) was used, with 100 to 200 ms being commonly used values in the assessment of VF in children Long saccadic latencies that vary with age have been reported in infancy.^31^^,^^32^ A further investigation of the effect of stimulus duration and saccade response time detection limits would, therefore, be of interest. A direct, standardized comparison against manual, operator-driven assessments would also provide insight into the potential benefits of SVOP in terms of accuracy, user experience, and test duration. Although a small number of repeated test attempts seem not to impede test performance, the current limiting requirement to restart the test prolongs the procedure unnecessarily. Revising the software and introducing the ability to pause and resume a test, only assessing the points not tested in previous attempts, may enable a gradual assessment of more descriptive test patterns that are less influenced by the infant's distraction and fatigue. Although other groups have used eye tracking to facilitate forms of limited VF assessment in children,^33^^–^^35^ to date we are not aware of any study that has included infants.

We have demonstrated the feasibility of SVOP assessment in normal infants. In children, we have obtained reliable results in normal children,^21^ children with brain tumours,^1^^,^^21^ and children with localized cerebral infarcts.^21^ Testing has proved more problematic in children with more widespread cerebral abnormalities. Tailor et al.,^35^ using slightly different technology, also found that SVOP performance was better in children with isolated visual pathway lesions than in children with more diffuse neurodisability. SVOP requires the presence of a number of intact sensory, integrative and motor neural systems. Although we anticipate that SVOP testing may be feasible in infants with localized cerebral abnormalities, it is likely that testing will prove less successful in infants with more widespread cerebral abnormalities.

This study has shown that modified SVOP is a feasible method for VF assessment in infants. Using a larger test stimulus, introducing a tiered test strategy, and using an animation fixation target enabled screening test patterns of up to 40 points. With further development, automated eye-tracking perimetry to explore visual function in infants and young children may prove feasible.
